# Age-dependent sex difference of non-alcoholic fatty liver disease in TSOD and *db/db* mice

**DOI:** 10.1371/journal.pone.0278580

**Published:** 2022-12-14

**Authors:** Erdenetsogt Dungubat, Hiroyuki Kusano, Ichiro Mori, Hirosuke Tawara, Mitsuko Sutoh, Naoki Ohkura, Masakatsu Takanashi, Masahiko Kuroda, Naoki Harada, Emiko Udo, Masakazu Souda, Bungo Furusato, Toshio Fukusato, Yoshihisa Takahashi

**Affiliations:** 1 Department of Pathology, School of Medicine, International University of Health and Welfare, Narita, Japan; 2 Department of Pathology, School of Biomedicine, Mongolian National University of Medical Sciences, Ulaanbaatar, Mongolia; 3 Institute for Animal Reproduction, Kasumigaura, Japan; 4 Faculty of Pharma Sciences, Laboratory of Host Defence, Teikyo University, Tokyo, Japan; 5 Department of Molecular Pathology, Tokyo Medical University, Tokyo, Japan; 6 Department of Applied Biological Chemistry, Graduate School of Agriculture, Osaka Metropolitan University, Osaka, Japan; 7 Clinical Genomics Center, Nagasaki University Hospital, Nagasaki, Japan; 8 General Medical Education and Research Center, Teikyo University, Tokyo, Japan; Tokyo University of Agriculture, JAPAN

## Abstract

According to previous clinical studies, the prevalence of non-alcoholic fatty liver disease (NAFLD) is higher in men than women only during the reproductive age. Animal models of NAFLD that reflect sex differences in humans have not been established. In this study, we examined sex differences in the hepatic lesions of Tsumura Suzuki obese diabetes (TSOD) and *db/db* mice, which are representative genetic models of NAFLD. Male and female TSOD and *db/db* mice were fed with a normal diet and tap water *ad libitum*. Six male and female mice of each strain were sacrificed at the ages of 3 and 9 months, respectively, and serum biochemical, pathological, and molecular analyses were performed. Serum aspartate aminotransferase (AST) levels were significantly higher in male than female mice of both strains at the age of 3 months; however, at 9 months, significant sex differences were not observed. Similarly, alanine aminotransferase (ALT) levels were significantly higher in male mice than in female TSOD mice at the age of 3 months; however, at 9 months, significant sex differences were not observed. Image analysis of histological slides revealed that the frequency of the steatotic area was significantly higher in male than female *db/db* mice at the age of 3 months; however, significant sex differences were not observed at 9 months. The frequency of Sirius red-positive fibrotic area was significantly higher in male than female mice in both strains at the age of 3 months; however, significant sex differences were not observed at 9 months. Serum AST and ALT levels and hepatic steatosis and fibrosis in TSOD and *db/db* mice showed age-dependent sex differences consistent with those observed in human NAFLD. These mice may be suitable for studying sex differences of the disease.

## Introduction

The accumulation of fat in hepatocytes (steatosis) in the absence of significant alcohol intake is called non-alcoholic fatty liver disease (NAFLD). Non-alcoholic fatty liver disease includes a spectrum of liver diseases ranging from simple steatosis (non-alcoholic fatty liver) to non-alcoholic steatohepatitis (NASH), which can progress to liver cirrhosis and hepatocellular carcinomas (HCC) [1–3]. It is a hepatic manifestation of metabolic syndrome and constitutes a major health concern worldwide, with an estimated prevalence of 25% in the general population [4] and 43–70% in patients with type 2 diabetes [5].

Previous clinical and epidemiological studies have suggested that there are sex differences in NAFLD in humans. During the reproductive age, the prevalence of NAFLD is higher in men than in women; however, following menopause, the prevalence in women becomes similar to or surpasses that in men [6–10]. Changes in estrogen levels are thought to be an important cause, but the detailed molecular mechanisms underlying age-dependent sex differences in human NAFLD have not yet been elucidated.

Animal models are useful for elucidating pathogenic mechanisms and developing new therapies for NAFLD/NASH. Ideal animal models should recapitulate all features of human diseases. In particular, animal models that accurately reflect sex differences in human NAFLD must be established to elucidate the mechanisms regulating sex differences and develop new therapies based on them. To date, several studies have examined sex differences in animal models of NAFLD/NASH; however, the results have been conflicting. In some studies, hepatic lesions were more severe in males than in females [11–14]; however, in other studies, the opposite was found [15,16] or there was no obvious sex difference [17]. The major problem with most of these studies is that they did not examine age-dependent changes that are characteristic of sex differences in NAFLD in humans. To the best of our knowledge, there has been only one study in which age-dependent changes were examined, but age-dependent changes in humans were not evident in the study [18]. Therefore, animal models of NAFLD that reflect sex differences in humans have not yet been established.

Tsumura Suzuki obese diabetes (TSOD) mice are a polygenic model of metabolic syndrome and spontaneously develop diabetes mellitus, obesity, glucosuria, hyperglycemia, and hyperinsulinemia. They exhibit a liver histopathology that mimics human NASH and eventually develop liver tumors, including HCC [19,20]. *Db/db* mice possess a natural mutation in the leptin receptor gene and exhibit obesity, insulin resistance, diabetes, and hepatic steatosis [21,22]. Currently, both TSOD and *db/db* mice are regarded as representative animal models of NAFLD. As these mice reflect many pathophysiological aspects of human NAFLD, we hypothesized that they might also reflect sex differences in human NAFLD. Therefore, in this study, we examined age-dependent sex differences in hepatic lesions of TSOD and *db/db* mice. In addition, significant molecules associated with sex differences in the livers of mice with NAFLD were investigated using real-time reverse transcription polymerase chain reaction (RT-PCR).

## Materials and methods

### Animals and experimental protocol

Twelve male and 12 female TSOD and *db/db* mice were each prepared at the Institute for Animal Reproduction (IAR) (Kasumigaura, Japan). They were kept in an animal experiment laboratory at IAR with free access to a normal diet (MF; Oriental Yeast, Tokyo, Japan) and tap water *ad libitum*. The conditions were as follows: 25°C, 45% humidity, and a 12 h light/12 h dark cycle. Six male and six female mice of each strain were sacrificed at the ages of 3 and 9 months, respectively, by collecting blood samples from the inferior vena cava (IVC) under deep anesthesia by isoflurane. Serum was separated by centrifugation (3000 rpm/10 min). The mice were starved for 17 h before sacrifice, and blood glucose levels, using blood from the tail vein, were measured by glucose analysis equipment (StatStrip Xpress 900; Nova Biomedical, Boston, MA, USA) immediately before sacrifice. Body weight was measured simultaneously. After blood was collected from the IVC, the liver of each mouse was extirpated and weighed, and samples for histological analysis, RNA purification, and snap-freezing were collected. This study was carried out in strict accordance with the recommendations in the Guide for the Care and Use of Laboratory Animals of the National Institutes of Health. The experimental protocol was approved by the Ethics Committee of IAR (on Mar. 1, 2018; Permit No. N562) and International University of Health and Welfare (on Dec. 16, 2019; Permit No. 19009NA).

### Biochemical analysis of serum

Serum aspartate aminotransferase (AST), alanine aminotransferase (ALT), total cholesterol (T-Cho), and triglyceride (TG) levels were analyzed using a Hitachi 7180 autoanalyzer (Hitachi High-Technologies Corporation, Tokyo, Japan) according to the manufacturer’s instructions. Serum insulin and adiponectin levels were measured by enzyme-linked immunosorbent assay (ELISA) using the ultrasensitive mouse insulin measuring kit (Morinaga Institute of Biological Science, Yokohama, Japan) and mouse/rat adiponectin ELISA kit (Otsuka Pharmaceutical, Tokyo, Japan), respectively. The homeostasis model assessment insulin resistance (HOMA-R), an index of insulin resistance, was calculated as follows: HOMA-R = fasting blood glucose (mg/dL) × fasting blood insulin (μU/mL) / 405. Further, serum levels of C-reactive protein (CRP), tumor necrosis factor (TNF)-α, and fibroblast growth factor (FGF) 21 were measured by ELISA using the mouse C-reactive protein (CRP) AssayMax ELISA kit (Assaypro, St. Chales, MO, USA), LBIS human TNF-α ELISA kit (FUJIFILM Wako Shibayagi Corporation, Shibukawa, Japan), and FGF-21 ELISA kit (BioVendor Laboratory Medicine, Brno, Czech Republic), respectively.

### Histopathological analysis

For histological analysis, the central parts of the two large liver lobes of each mouse were cut along the long axis, and 3 mm-thick liver tissue was taken from each lobe. Therefore, two liver tissue samples were collected from each mouse. The tissues were fixed in 10% neutral-buffered formalin and embedded in paraffin. Sections (3–4 μm-thick) were made from each liver tissue, and hematoxylin and eosin (H&E) and Sirius red staining were performed to evaluate hepatic histopathology, including fibrosis. Therefore, the largest cut surfaces of the two large liver lobes were used to generate histological slides for each mouse (two sections per mouse). Both sections of all mice were used for staining and evaluated in all histological examinations (including image analysis). Histopathology was evaluated semi-quantitatively using the scoring validated by Kleiner et al. [23]. Detailed scoring criteria for steatosis, lobular and portal inflammation, and hepatocellular ballooning have been described in our previous studies [24,25]. The method for calculating the NAFLD activity score (NAS) and staging of fibrosis was also described in our previous papers [24,25]. The histological slides were observed by three hepatic pathologists (E.D., T.F., and Y.T.). Often, each pathologist’s evaluation showed good concordance. When there were minor differences in the evaluation, the three pathologists discussed and made the final decision.

### Whole-slide quantitative image analysis

We performed whole-slide quantitative image analysis to evaluate hepatic steatosis and fibrosis using previously validated methods [26,27]. All H&E- and Sirius red-stained slides were scanned at 40× resolution using a digital slide scanner NanoZoomer S210 (Hamamatsu Photonics, Hamamatsu, Japan). Digital images of the entire liver sections were captured using NDP.scan v3.3 software (Hamamatsu Photonics). The prevalence of macro/micro lipid vesicles in hepatocytes and Sirius red-positive fibrotic areas in these whole-slide images were analyzed using the open-source pathology image analysis software QuPath v0.2.0 (University of Edinburgh, Edinburgh, UK). The representative tissue areas were annotated using manual tools, and the pixel classifier (artificial neural network MLP) was trained on annotations. Once the tissue classifier was trained, the algorithms were applied to all the images, and the steatosis and fibrosis areas (%) were computed as the proportion to the classified tissue.

### Terminal deoxynucleotidyl transferase dUTP nick end labeling (TUNEL) staining

To evaluate apoptotic hepatocytes, we performed TUNEL staining using the DeadEnd colorimetric TUNEL system (Promega, Madison, WI, USA) according to the manufacturer’s instructions. Entire histological slides were observed, and the total number of positively stained hepatocytes was counted for each mouse.

### Hepatic levels of TG and T-Cho

We measured TG and T-Cho levels in the liver biochemically using approximately 50 mg of frozen tissue from each mouse. The detailed methods were described in our previous paper [25].

### Real-time RT-PCR assays

Total RNA (50 ng/sample) was isolated from mouse liver specimens using ISOGEN (Nippon Gene, Tokyo, Japan) or RNAzol RT Reagent (Molecular Research Center, Cincinnati, OH, USA) and then reverse transcribed using Oligo (dT) primers and the Transcriptor First Strand cDNA Synthesis Kit (Roche Diagnostics, Basel, Switzerland) according to the manufacturer’s recommendations. Quantitative real-time PCR was performed using 5 μL of 20-fold diluted cDNA in a final volume of 20 μL using the FastStart Universal Probe Master Mix (Roche Diagnostics). The sequences of primers and probes are listed in Table 1. Data were analyzed using Light Cycler 96 Software Ver.1.1 (Roche Diagnostics GmbH, Basel, Switzerland). The comparative Ct method was used for the relative quantitation of samples.

**Table 1:** Primer sequences and probes used for real-time reverse transcription polymerase chain reaction.

Genes	Forward primers	Reverse primers	Probes*
*PPARα*	5’-cacgcatgtgaaggctgtaa-3’	5’-gctccgatcacacttgtcg-3’	#41 (cat.no. 04688007001)
*PPARγ*	5’-gaaagacaacggacaaatcacc-3’	5’-gggggtgatatgtttgaacttg-3’	#7 (cat. no. 04685059001)
*MYD88*	5’-gaggatatactgaaggagctgaagtc-3’	5’-cctggttctgctgcttacct-3’	#101 (cat. no. 04692195001)
*FGF21*	5’-agatggagctctctatggatcg-3’	5’-gggcttcagactggtacacat-3’	#67 (cat. no. 04688660001)
*TNF-α* (1)	5’-ctgtagcccacgtcgtagc- 3’	5’-ttgagatccatgccgttg-3’	#25 (cat. no. 04686993001)
*TNF-α* (2)	5’-tcttctcattcctgcttgtgg-3’	5’-ggtctgggccatagaactga-3’	#49 (cat. no. 04688104001)
*IL-6* (1)	5’-gctaccaaactggatataatcagg-3’	5’-ccaggtagctatggtactccagaa-3’	#6 (cat. no. 04685032001)
*IL-6* (2)	5’-acaaagccagagtccttcaga-3’	5’-tggtccttagccactccttc-3’	#78 (cat. no. 04688660001)

* Roche Diagnostics. Β-actin: Universal Probe Library Mouse ACTB Gene Assay (Sigma-Aldrich, 05046190001). PPAR, peroxisome proliferator-activated receptor; MYD, myeloid differentiation primary response; FGF, fibroblast growth factor; TNF, tumor necrosis factor; IL, interleukin.

To examine the mechanisms underlying sex differences in these NAFLD model mice in more depth, we additionally performed real-time RT-PCR assays for genes associated with inflammation (*CD11b*, *CD11c*, *F4/80*, *interleukin (IL)-1b*, and *IL-8*). The assay ID of each gene was as follows: *CD11b*, Mm00434455_m1; *CD11c*, Mm00498701_m1; *F4/80*, Mm00802529_m1; *IL-1b*, Mm00434228_m1; *IL-8*, Mm00441263_m1; *beta-actin* (internal control gene), Mm00607939_s1. Since expression of the genes examined was not detectable for many samples, we performed further additional real-time RT-PCR assays for genes associated with lipid metabolism (*acetyl-CoA carboxylase (ACC)*, *CD36*, *fatty acid synthase (FASN)*, *carnitine palmitoyltransferase (CPT) 1A*, and *sterol regulatory element-binding protein (SREBP)-1c*) using the same method as in our previous study. The detailed procedures including primer sequences were described in our previous paper [28]. However, we used different primers for *SREBP-1c*, and the sequences were as follows: forward, 5’-AGCTGTCGGGGTAGCGTCTG-3’; reverse, 5’- GAGAGTTGGCACCTGGGCTG-3’. The *beta-actin* gene was used as the internal control and the primer sequences were as follows: forward, 5’-TTGCTGACAGGATGCAGAAG-3’; reverse, 5’-GTACTTGCGCTCAGGAGGAG-3’.

### Statistical analysis

Continuous variables are presented as the mean ± standard deviation (SD). A one-tailed or two-tailed t-test was performed to evaluate the significance of the differences since the comparison was made between two groups (male and female) and normal distribution could be assumed for these continuous data. A one–tailed or two-tailed test was appropriately selected based on the null hypothesis that male predominance of NAFLD and its associated metabolic disorders is observed only in young mice. For semi-quantitative data in the histological evaluation, data are presented as median (min. to max.). The Mann-Whitney U test was used to determine statistical significance since the comparison was made between two groups and normal distribution could not be assumed for these categorical data. Outliers confirmed by the Smirnov-Grubbs’s test or Thompson’s test were removed since these are generally the most accepted outlier tests. Statistical analyses were performed using BellCurve for Excel, Version 3.21 (Social Survey Research Information, Tokyo, Japan). Statistical significance was set at a p value < 0.05.

## Results

### General observations

Table 2 provides data regarding body weight, liver weight, and the liver-to-body weight ratio. The body and liver weights of male TSOD mice at 3 months were significantly higher than those of age-matched females (p < 0.001 for both); however, no significant difference in the liver-to-body weight ratio was observed. There was no significant sex difference in 3-month-old *db/db* mice with respect to body and liver weights or liver-to-body weight ratio. The liver weight and liver-to-body weight ratio of 9-month-old TSOD mice were significantly higher in males than in females (p = 0.012 and 0.031, respectively). The body and liver weights of male *db/db* mice at the age of 9 months were significantly higher than those of age-matched females (p = 0.001 for both); however, no significant difference in the liver-to-body weight ratio was observed.

**Table 2:** Body and liver weights of the mice.

	TSOD mice		*db/db* mice	
	Male	Female	Male	Female
3-month-old				
Body weight (g)	51.6 ± 3.0	36.1 ± 1.6*	44.1 ± 0.3	44.0 ± 1.8
Liver weight (g)	1.9 ± 0.2	1.3 ± 0.0*	2.5 ± 0.2	2.2 ± 0.4
Liver/body weight ratio (%)	3.6 ± 0.0	3.6 ± 0.1	5.7 ± 0.4	5.1 ± 0.7
9-month-old				
Body weight (g)	58.3 ± 3.0	55.1 ± 4.4	51.9 ± 1.0	44.2 ± 3.6*
Liver weight (g)	2.3 ± 0.3	1.8 ± 0.2*	2.6 ± 0.3	2.0 ± 0.1*
Liver/body weight ratio (%)	3.9 ± 0.4	3.3 ± 0.4*	5.1 ± 0.5	4.6 ± 0.4

Data are presented as the mean ± SD.

*Significantly different from male mice (p < 0.05).

### Serum biochemical data

Table 3 shows the serum data corresponding to each mouse strain at 3 and 9 months of age. Serum AST levels were significantly higher in 3-month-old male TSOD and *db/db* mice than in their female counterparts (p = 0.009 and 0.022, respectively). However, at 9 months, no significant sex difference was observed in either strain. ALT levels were significantly higher in 3-month-old male TSOD mice than their female counterparts (p = 0.017); however, at 9 months, significant sex differences were not observed. T-Cho levels were significantly higher in male TSOD mice than in female TSOD mice at 3 months (p < 0.001), and they were significantly higher in 9-month-old male TSOD and *db/db* mice than in their female counterparts (p = 0.003 and 0.041, respectively). At 3 and 9 months, TG levels were significantly higher in male TSOD mice than in females (p = 0.031 and 0.036, respectively). Glucose levels in the tail vein blood were significantly higher in 3-month-old male *db/db* mice than their female counterparts (p = 0.001), but they were significantly higher in 9-month-old female TSOD and *db/db* mice than in males (p = 0.014 and 0.025, respectively). Insulin levels were significantly higher in 3-month-old male TSOD mice than in females (p = 0.014) and 9-month-old male TSOD and *db/db* mice than their female counterparts (p < 0.001 and p = 0.010, respectively). Adiponectin levels were significantly higher in female TSOD mice than in males at the ages of 3 and 9 months (p < 0.001 for both) but were significantly higher in male *db/db* mice than in females at the age of 3 months (p = 0.044). HOMA-R levels were significantly higher in male TSOD mice than female mice at 3 and 9 months of age (p = 0.039 and p < 0.001, respectively). CRP levels were significantly higher in 3-month-old male TSOD and *db/db* mice than in their female counterparts (p < 0.001 and p = 0.001, respectively) and significantly higher in 9-month-old male TSOD mice than in their female counterparts (p = 0.022). FGF21 levels were higher in female mice than in male mice in both age groups and strains, and a significant sex difference was observed in 9-month-old TSOD mice (p = 0.001). TNF-α was undetectable in all samples suggesting very low serum levels.

**Table 3:** Biochemical data of serum.

	TSOD mice		*db/db* mice	
	Male	Female	Male	Female
3-month-old				
AST (IU/L)	337.7 ± 124.9	187.8 ± 37.1*	124.2 ± 23.2	97.3 ± 16.5*
ALT (IU/L)	129.2 ± 38.7	85.2 ± 5.8*	110.3 ± 43.7	76.0 ± 26.7
T-Cho (mg/dL)	378.2 ± 47.4	244.0 ± 14.7*	199.0 ± 25.4	182.0 ± 23.2
TG (mg/dL)	484.0 ± 86.2	391.8 ± 64.9*	368.3 ± 129.0	417.7 ± 122.7
Glucose (mg/dL)	74.8 ± 10.0	80.3 ± 16.5	278.2 ± 43.2	162.2 ± 46.8*
Insulin (ng/mL)	1.2 ± 0.4	0.7 ± 0.2*	4.0 ± 2.0	5.8 ± 3.5
Adiponectin (μg/mL)	10.9 ± 0.9	23.2 ± 1.9*	27.4 ± 6.5	20.8 ± 2.7*
HOMA-R	5.2 ± 0.8	3.8 ± 1.4*	72.1 ± 37.0	54.2 ± 22.6
CRP (μg/mL)	4.4 ± 0.1	2.5 ± 0.2*	13.2 ± 0.5	11.9 ± 0.5*
FGF21 (pg/mL)	2460.6 ± 1056.2	3427.3 ± 1041.3	100.0 ± 104.6	234.1 ± 300.8
9-month-old				
AST (IU/L)	462.4 ± 52.3	384.8 ± 186.4	189.7 ± 92.1	103.3 ± 17.5
ALT (IU/L)	234.7 ± 51.6	177.8 ± 68.6	222.3 ± 128.7	96.2 ± 32.5
T-Cho (mg/dL)	302.0 ± 45.3	216.5 ± 26.0*	177.5 ± 30.2	144.8 ± 16.1*
TG (mg/dL)	401.7 ± 72.0	322.8 ± 34.7*	222.5 ± 120.2	243.8 ± 75.6
Glucose (mg/dL)	64.7 ± 13.4	83.8 ± 4.3*	148.5 ± 59.0	240.8 ± 62.8*
Insulin (ng/mL)	2.6 ± 0.8	0.6 ± 0.2*	2.0 ± 0.4	1.2 ± 0.4*
Adiponectin (μg/mL)	8.3 ± 2.3	22.3 ± 2.8*	9.8 ± 1.6	10.6 ± 1.9
HOMA-R	10.5 ± 2.5	3.2 ± 1.0*	20.9 ± 5.4	18.0 ± 6.1
CRP (μg/mL)	4.0 ± 0.9	2.9 ± 0.4*	11.6 ± 1.9	10.8 ± 0.3
FGF21 (pg/mL)	3223.7 ± 898.2	6408.2 ± 1177.7*	773.7 ± 383.2	1064.1 ± 131.2

Data are presented as the mean ± SD.

*Significantly different from male mice (p < 0.05). AST, aspartate aminotransferase; ALT, alanine aminotransferase; T-Cho, total cholesterol; TG, triglyceride; HOMA-R, homeostasis model assessment insulin resistance; CRP, C-reactive protein; FGF, fibroblast growth factor.

### Histopathological findings

**Fig 1:**
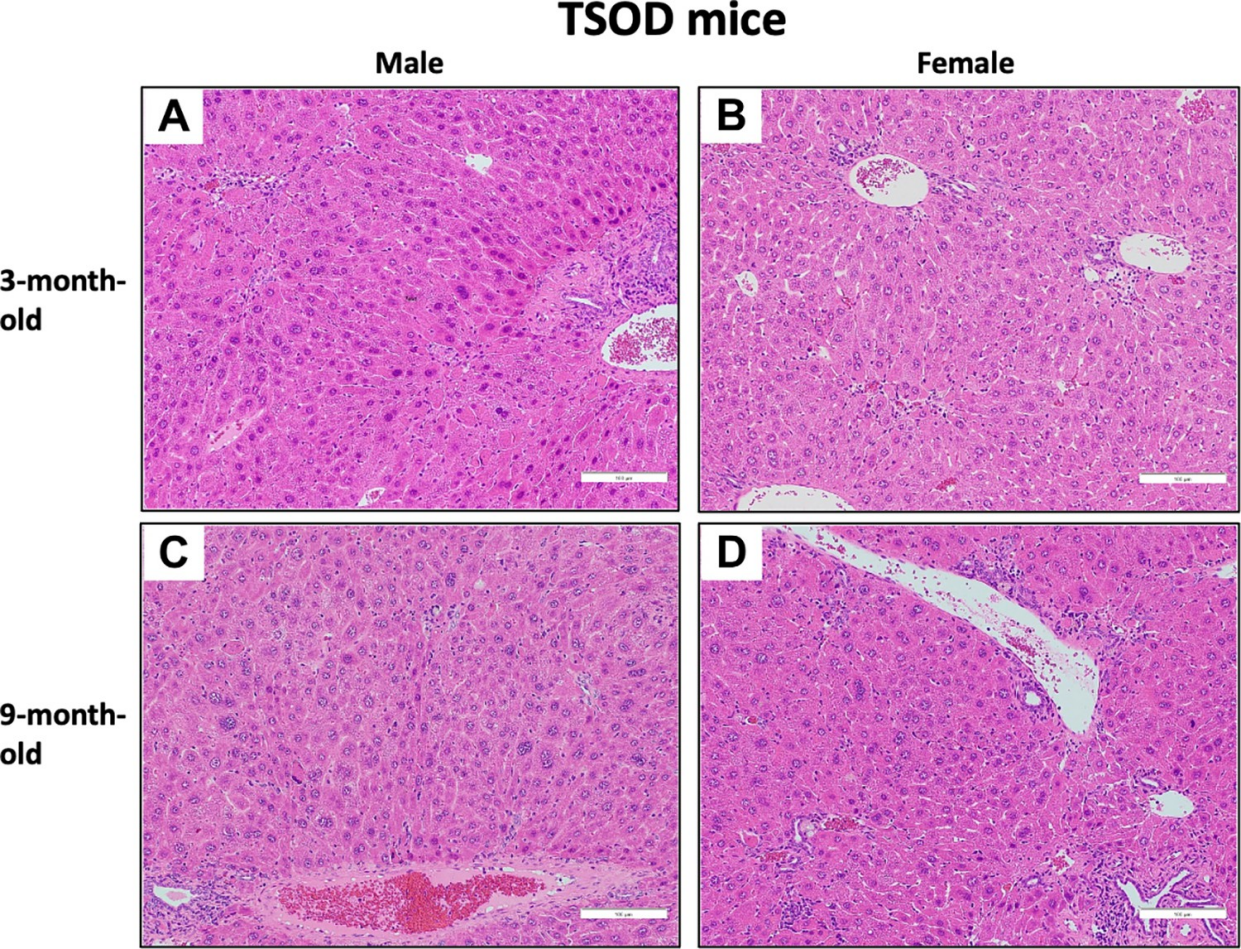
Photomicrographs of the liver of TSOD mice at the ages of 3 and 9 months. **A-D)** In male and female TSOD mice, intralobular and portal inflammation is observed, although hepatic steatosis and ballooning are not conspicuous. Intralobular inflammation tends to be more severe in male mice than in females at 3 months. (H&E staining, original magnification ×200).

**Fig 2:**
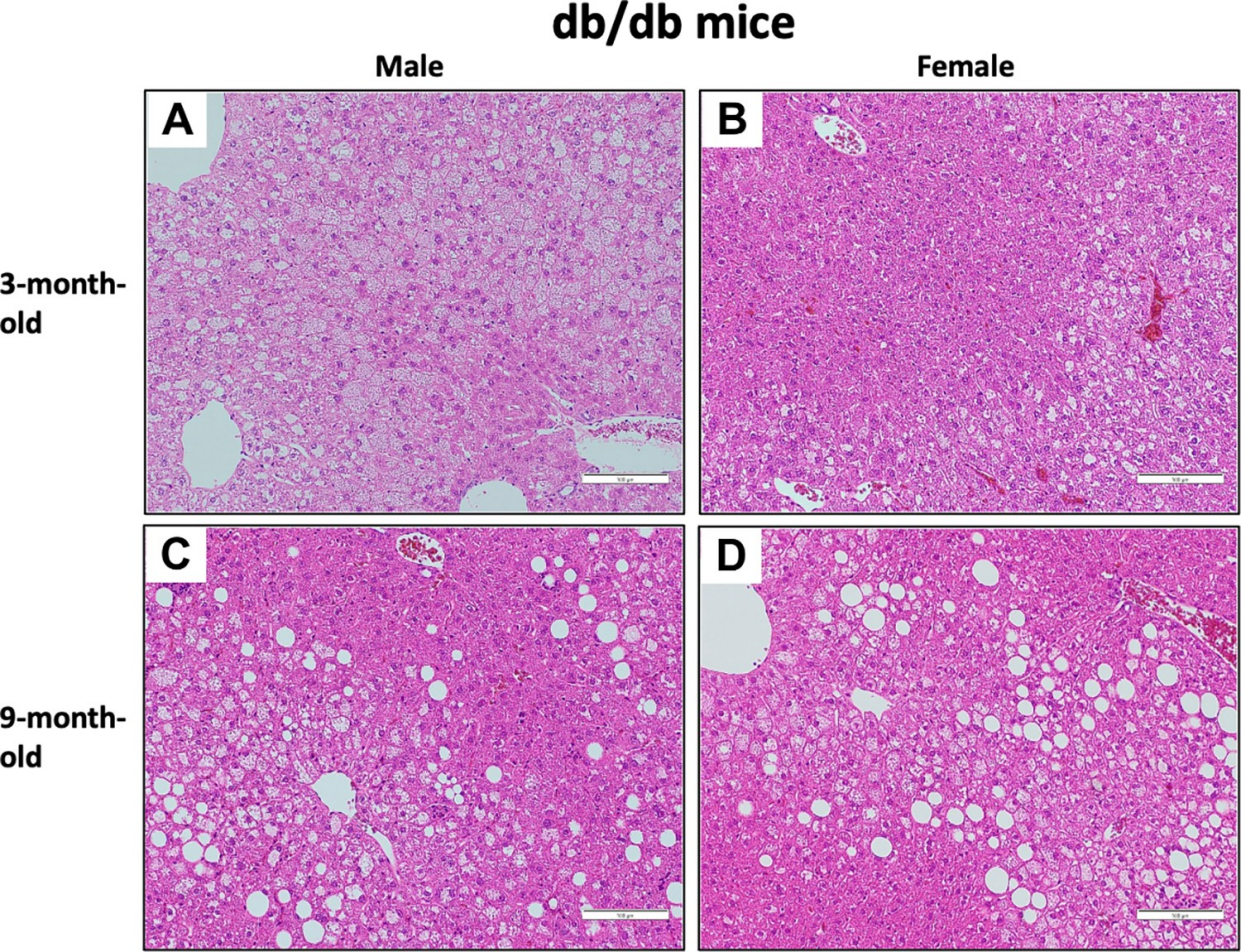
Photomicrographs of the liver of *db/db* mice at 3 and 9 months. **A-D)** In *db/db* mice, steatosis is conspicuous in both 3- and 9-month-old mice. Intralobular and portal inflammation is milder compared to that in TSOD mice, and ballooning is not conspicuous at both ages. Steatosis is more severe in male mice than females at 3 months. (H&E staining, original magnification ×200).

With respect to liver histopathology, intralobular and portal inflammation was observed in male and female TSOD mice, although hepatic steatosis and ballooning were not conspicuous. Intralobular inflammation tended to be more severe in male mice than female mice at the age of 3 months (Fig 1). In *db/db* mice, steatosis was conspicuous in both the 3- and 9-month-old mice. Intralobular and portal inflammation was milder than in TSOD mice, and ballooning was not conspicuous at either age. Steatosis was more severe in male mice than female mice at 3 months (Fig 2). In the semi-quantitative analysis, steatosis grade was significantly higher in male mice than female *db/db* mice at 3 months (p = 0.027); however, significant sex differences were not observed at 9 months. Intralobular inflammation in 3-month-old TSOD mice tended to be more severe in male mice than females (p = 0.058) (Table 4).

**Table 4:** Histopathological findings.

	TSOD mice		*db/db* mice	
	Male	Female	Male	Female
3-month-old				
Steatosis grade	0 (0–0)	0 (0–0)	3 (2–3)	2 (2–3)*
Intralobular inflammation	2.5 (1–3)	1 (1–2)	0.5 (0–1)	0 (0–1)
Portal inflammation	1 (1–2)	1 (1–2)	0 (0–0)	0 (0–0)
Ballooning	0 (0–0)	0 (0–0)	0 (0–0)	0 (0–0)
NAS	2.5 (1–3)	1 (1–2)	3.5 (2–4)	2.5 (2–3)
Fibrosis stage	0 (0–0)	0 (0–0)	0 (0–0)	0 (0–0)
9-month-old				
Steatosis grade	0 (0–1)	0 (0–0)	2 (2–3)	2 (2–2)
Intralobular inflammation	2 (1–3)	2 (1–3)	1.5 (1–2)	1 (1–1)
Portal inflammation	2 (1–2)	2 (1–2)	0 (0–1)	0 (0–1)
Ballooning	0 (0–0)	0 (0–0)	0.5 (0–1)	0 (0–1)
NAS	2 (2–3)	2 (1–3)	4 (3–6)	3 (3–4)
Fibrosis stage	1 (0–1)	0 (0–1)	0.5 (0–1)	0 (0–1)

Data are presented as the median (min-max).

*Significantly different from male mice (p < 0.05). NAS, NAFLD activity score.

### Whole-slide quantitative image analysis of histological slides

**Fig 3:**
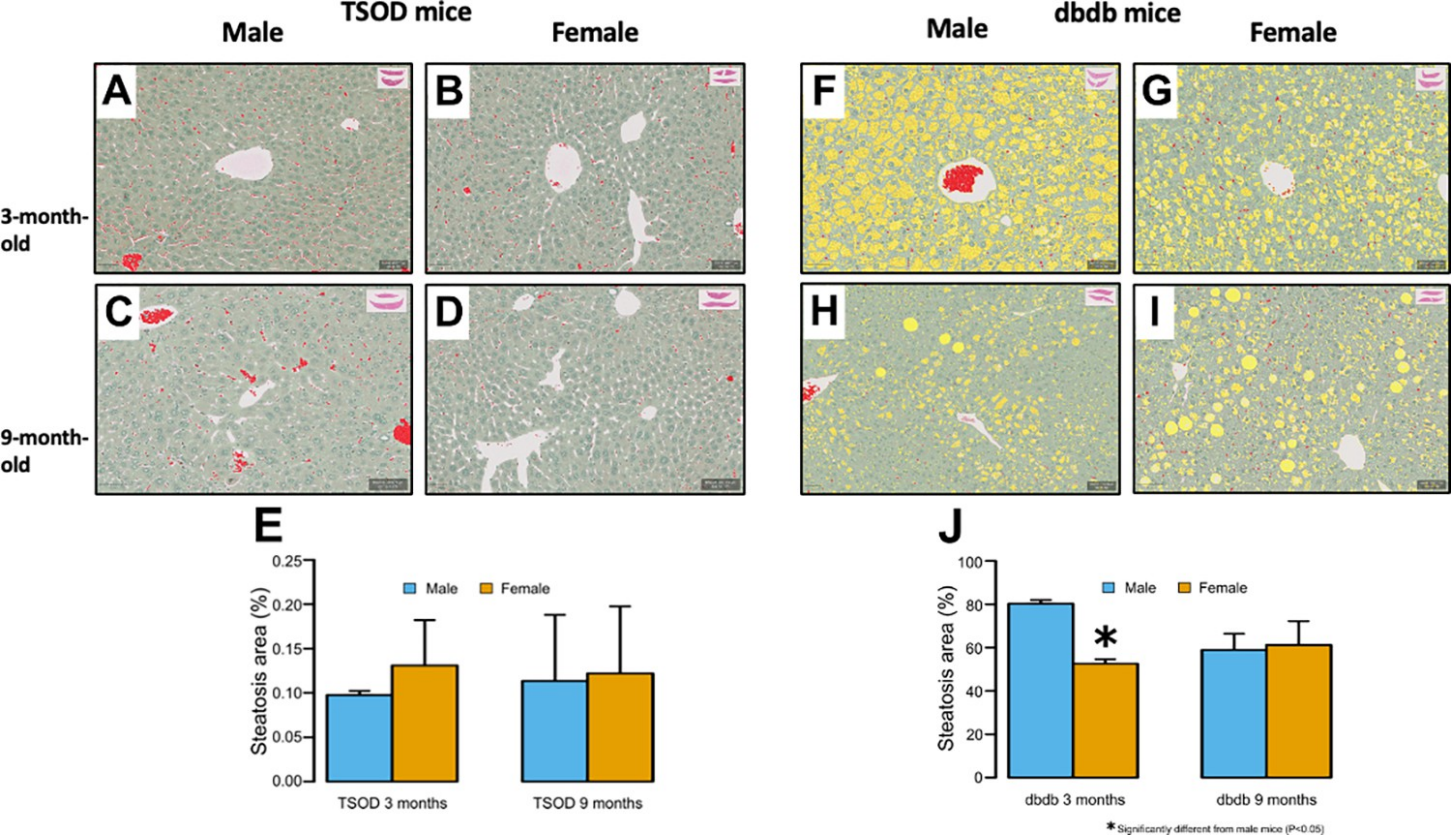
Image analysis for quantification of steatosis. **A-E)** There is no significant sex difference in the frequency of steatotic area in 3- and 9-month-old TSOD mice (one-tailed t-test for young mice and two-tailed t-test for old mice). **F-J)** The frequency of steatotic area is significantly higher in 3-month-old male *db/db* mice than females (one-tailed t-test); however, significant sex differences are not observed at 9 months (two-tailed t-test). (A-D,F-I: Original magnification ×400).

**Fig 4:**
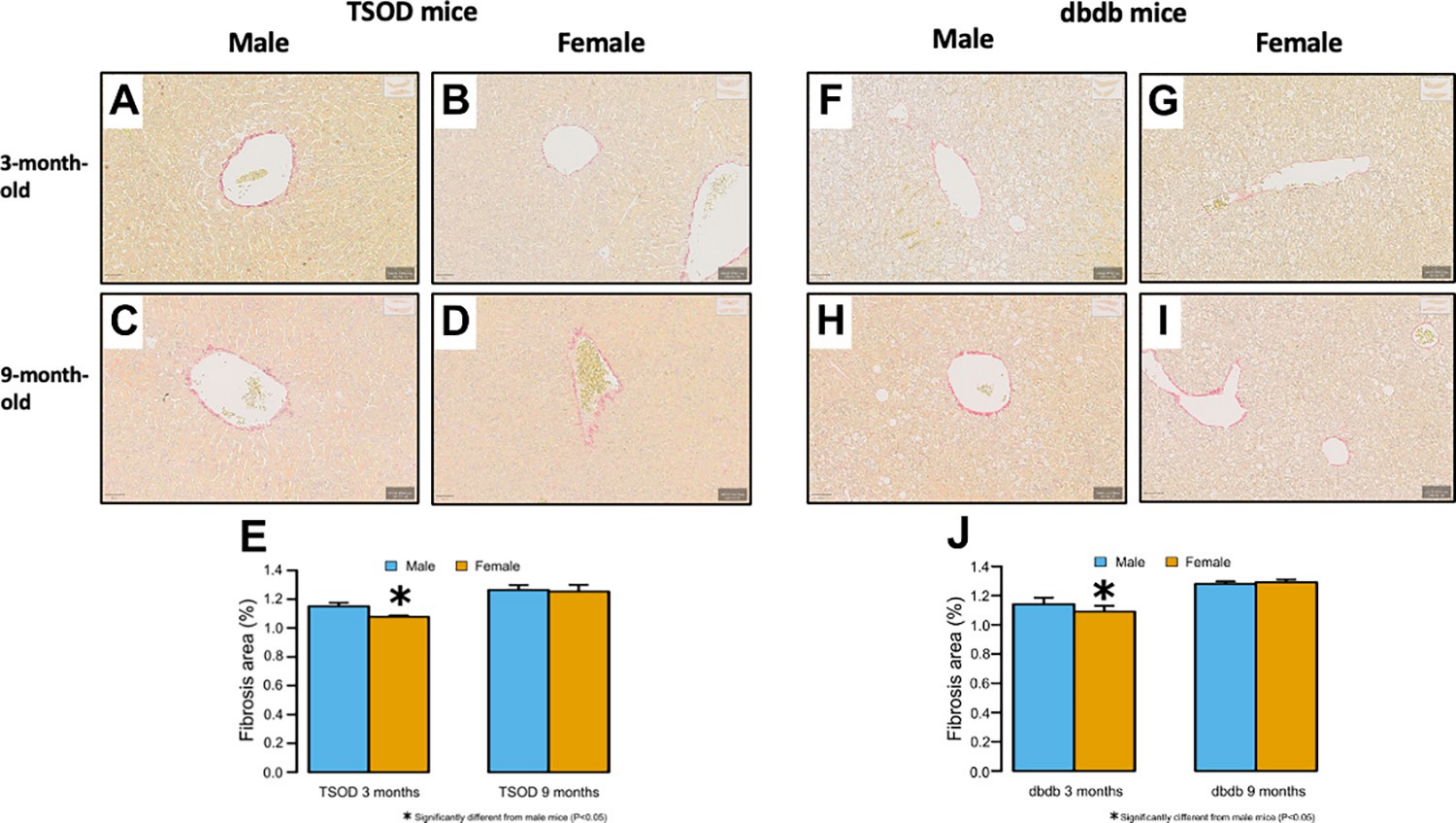
Image analysis for quantification of fibrosis. **A-J)** The frequency of Sirius red-positive fibrotic area is significantly higher in male mice than in female mice in TSOD and *db/db* mice at the age of 3 months (one-tailed t-test); however, significant sex differences are not observed in both strains at 9 months (two-tailed t-test). (A-D,F-I: Original magnification ×400).

To evaluate steatosis and fibrosis accurately and objectively, we performed whole-slide quantitative image analysis using H&E- and Sirius red-stained slides. The frequency of steatotic area was significantly higher in 3-month-old male *db/db* mice than in their female counterparts (p < 0.001); however, significant sex differences were not observed at 9 months (Fig 3). The frequency of Sirius red-positive fibrotic area was significantly higher in male TSOD and *db/db* mice than females at 3 months (p < 0.001 and p = 0.005, respectively); however, no significant sex difference was observed in either strain at 9 months (Fig 4).

### Apoptotic hepatocytes detected by TUNEL staining

**Fig 5:**
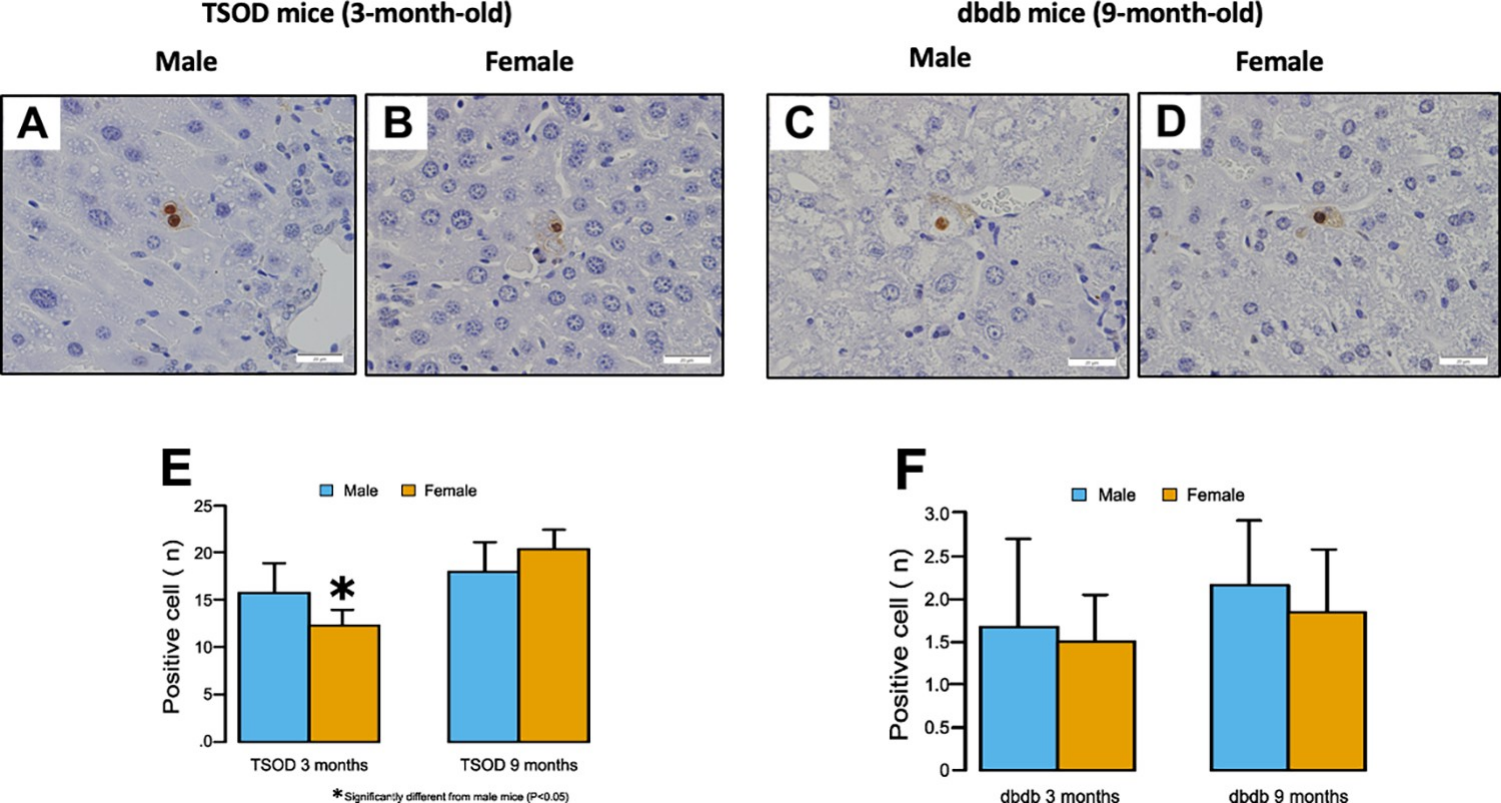
Terminal deoxynucleotidyl transferase dUTP nick end labeling (TUNEL) staining of liver tissues. **A-D)** Positively stained hepatocytes are observed in 3-month-old male (A) and female (B) TSOD mice and 9-month-old male (C) and female (D) *db/db* mice (original magnification ×400). **E)** The number of positively stained hepatocytes is significantly higher in 3-month-old male TSOD mice than in females (one-tailed t-test), but there is no significant sex difference in 9-month-old TSOD mice. **F)** For *db/db* mice, there is no significant sex difference in both age groups.

Fig 5 shows total number of TUNEL-positive apoptotic hepatocytes in each mouse. The number of positively stained hepatocytes was significantly higher in 3-month-old male TSOD mice than in females (p = 0.027), but there was no significant sex difference in 9-month-old TSOD mice. For *db/db* mice, there was no significant sex difference in both age groups.

### TG and T-Cho levels in the liver

**Fig 6:**
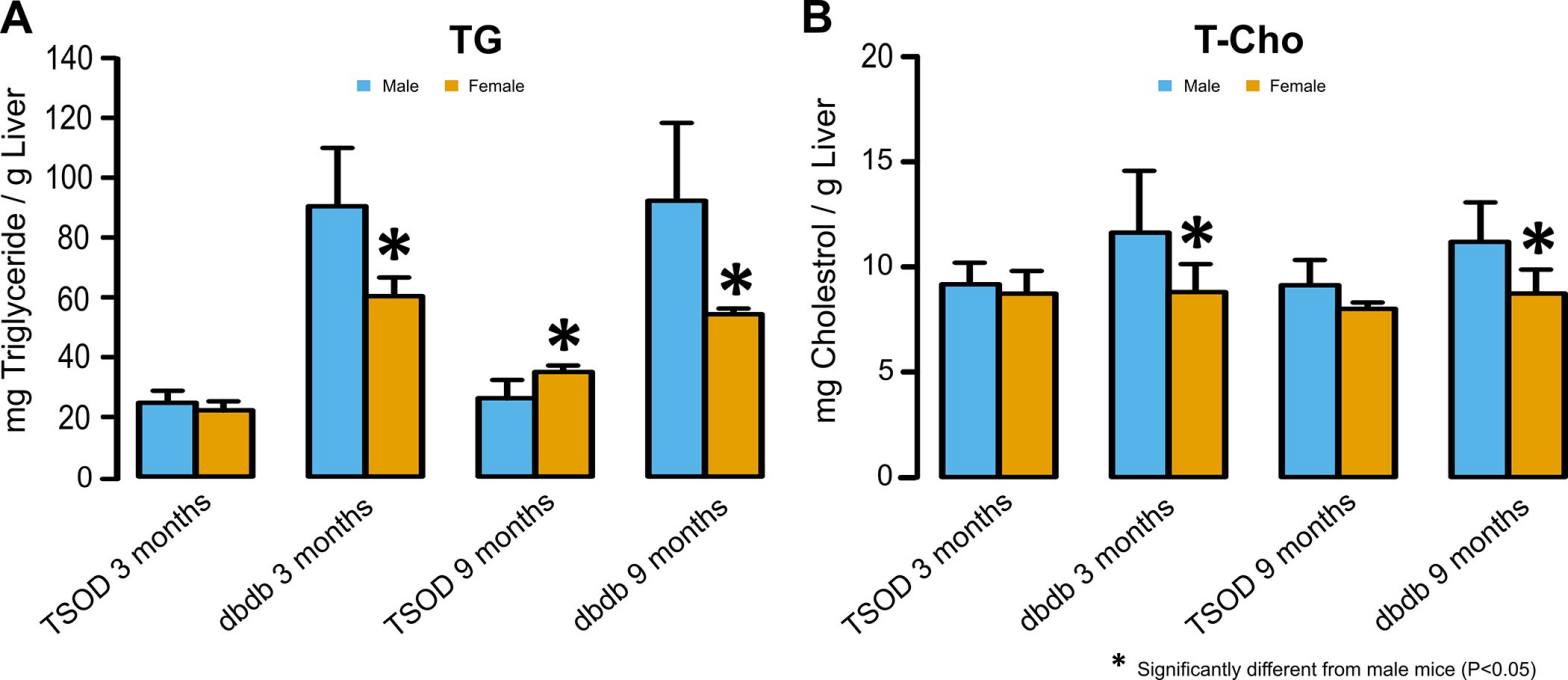
Triglyceride (TG) and total cholesterol (T-Cho) levels in the liver. **A)** At 3 months of age, TG levels in the livers of TSOD mice show no significant sex difference; however, at 9 months, they are significantly higher in females than in males (two-tailed t-test). For *db/db* mice, hepatic TG levels are significantly higher in male mice than in females at 3 and 9 months of age (one-tailed t-test for young mice and two-tailed t-test for old mice). **B)** T-Cho levels in the livers of male *db/db* mice are significantly higher than those in females at 3 and 9 months of age (one-tailed t-test for young mice and two-tailed t-test for old mice).

At 3 months of age, TG levels in the livers of TSOD mice showed no significant sex difference; however, at 9 months, they were significantly higher in females than in males (p = 0.025). For *db/db* mice, hepatic TG levels were significantly higher in male mice than in females at 3 and 9 months of age (p = 0.011 and 0.023, respectively). T-Cho levels in the livers of male *db/db* mice were significantly higher than those in females at 3 and 9 months of age (p = 0.037 and 0.021, respectively) (Fig 6).

### Gene expression in the liver

**Fig 7:**
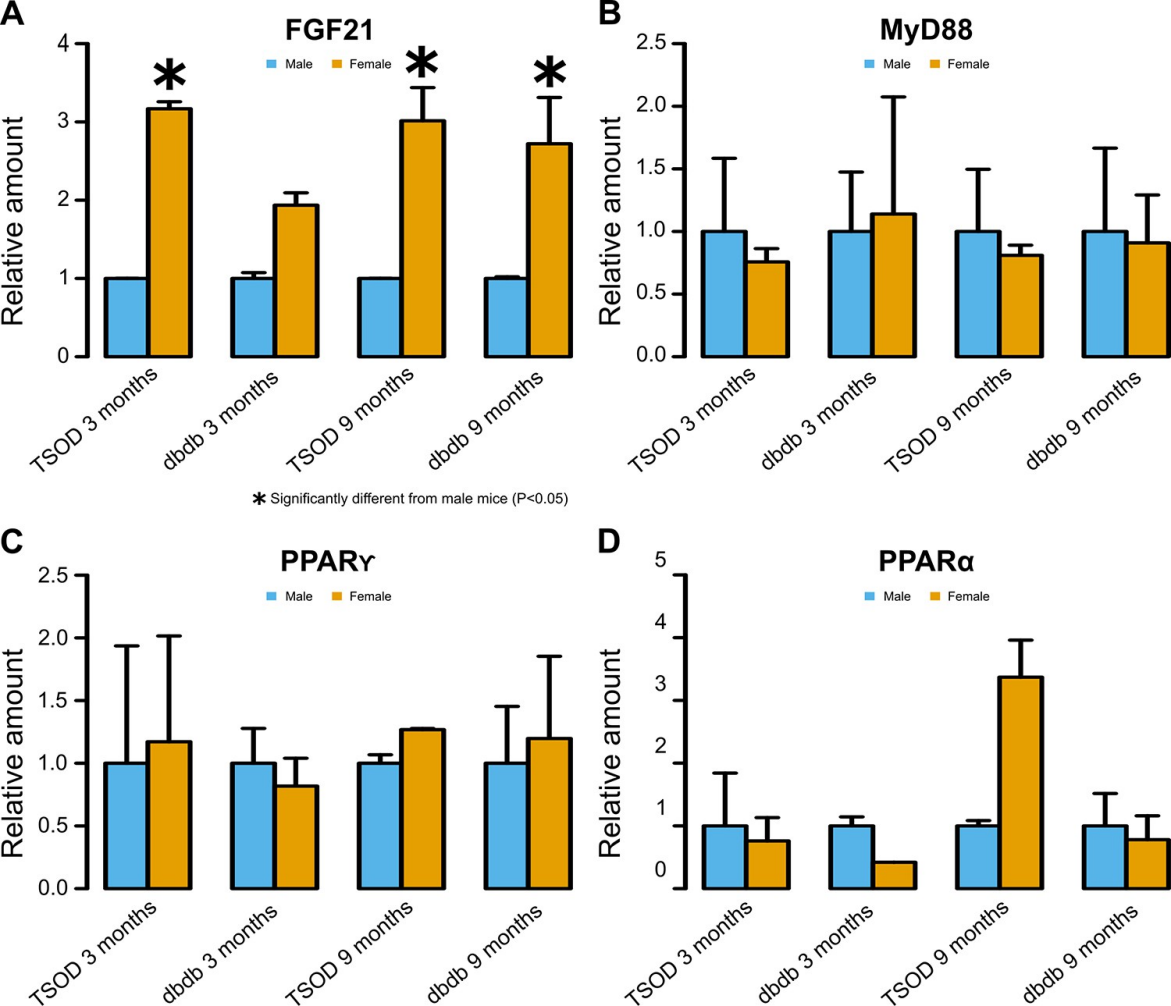
Hepatic expression levels of genes determined by real-time reverse transcription polymerase chain reaction. **A)** The expression levels of *fibroblast growth factor (FGF)21* mRNA are significantly higher in female TSOD mice than males at 3 months and significantly higher in female TSOD and *db/db* mice than males at 9 months (two-tailed t-test). **B-D)** For the expression levels of *myeloid differentiation primary response (MYD)88*, *peroxisome proliferator-activated receptor (PPAR)α*, and *PPARγ*, significant sex differences are not observed in both strains and age groups (two-tailed t-test).

The hepatic expression levels of *FGF21* mRNA, determined by real-time RT-PCR, were significantly higher in female TSOD mice than in males at 3 months (p = 0.003) and significantly higher in female TSOD and *db/db* mice than in males at 9 months (p = 0.003 and 0.021, respectively) (Fig 7A). For the expression levels of *myeloid differentiation primary response (MYD)88*, *peroxisome proliferator-activated receptor (PPAR)α*, and *PPARγ*, significant sex differences were not observed in either strain or age group (Fig 7B–7D). The expression of *TNF-α* and *IL-6* was not detectable, although two sets of primers were tried.

In the additional real-time RT-PCR analysis, the expression of genes examined was not detectable for many samples probably owing to deterioration of the sample quality after long-term preservation. Actually, the expression of *SREBP-1c*, *CD11b*, *CD11c*, and *IL-8* was not detectable for all samples. However, the expression of *ACC*, *CD36*, *FASN*, *CPA 1A*, *F4/80*, and *IL-1b* was detectable at least partially (S1 Fig). At 3 months of age, the expression levels of *FASN* were significantly higher in male TSOD mice than in females (p = 0.037), but they were significantly higher in female *db/db* mice than in males (p = 0.005). At 9 months of age, *FASN* expression showed no significant sex difference in both strains. Expression levels of *CPT 1A* were significantly higher in 9-month-old female TSOD mice than in their male counterparts (p = 0.009). For the expression levels of *ACC*, *CD36*, *F4/80*, and *IL-1b*, no significant sex difference was detected.

## Discussion

In this study, serum AST and/or ALT levels were significantly higher in 3-month-old male TSOD and *db/db* mice than in females; however, at the age of 9 months, there was no significant sex difference. Image analysis of liver histopathology revealed that steatosis and/or fibrosis were significantly more severe in 3-month-old male TSOD and *db/db* mice than their female counterparts; however, at the age of 9 months, there was no significant sex difference. These findings are consistent with sex differences observed in human NAFLD, with male predominance at a young age and equal prevalence or female predominance at an old age (especially after menopause). Serum ALT is considered important for determining the severity of NAFLD since it is a highly specific marker of liver cell damage. The correlation coefficients between serum ALT levels and body weight, between serum ALT and AST levels, and between serum ALT levels and NAS were 0.569, 0.690, and 0.353, respectively, suggesting weak-to-moderate and positive correlations.

Conflicting results have been reported in previous studies on sex differences in NAFLD animal models [11–17]. In studies reporting male predominance, methionine- and choline-deficient (MCD) diet [11,14], high fat diet [13], or hepatocyte-specific Pten-deficient mice models [12] were used. In contrast, in studies reporting female predominance, high-fructose [15] and cafeteria [16] diet models were used. The limitation of these studies is that they did not examine age-dependent changes. The sacrificial age was 9–40 weeks in studies reporting male predominance and 17–24 weeks in studies reporting female predominance; the animals were not necessarily young in studies reporting male predominance or old in studies reporting female predominance. To the best of our knowledge, only one study has examined age-dependent sex differences in a NAFLD animal model [18]. In the study, hepatic lesions of *p62/Sqstm1* and *Nrf2* double-knockout mice were examined at 8, 30, and 50 wk of age. In the study, inflammatory activity, extent of fibrosis, and serum AST levels were consistently higher in male mice than female mice. Sex differences in inflammatory activity were not statistically significant at 8 wk, but significant male predominance was observed at 30 and 50 wk. Thus, the model did not correctly reflect sex differences in human NAFLD. In the present study, serum AST and ALT levels and the extent of hepatic steatosis and fibrosis in TSOD and *db/db* mice basically reflected sex differences in human NAFLD; therefore, these mice models are thought to be more appropriate than *p62/Sqstm1* and *Nrf2* double-knockout mice for sex difference studies.

Here, we set the sacrificial age of young mice at 3 months and that of old mice at 9 months. We set the sacrificing age of young mice at 3 months because the sacrificing age is 3 months or older in most animal experiments on NAFLD. In contrast, we set the sacrificing age of old mice at 9 months because after this age: 1) liver tumors develop in TSOD mice, which may influence morphological and chemical findings in the liver [19]; and 2) *db/db* mice begin to die [29]. There are no detailed data on the reproductive ages of TSOD and *db/db* mice. However, in the IAR, male TSOD mice over 28 weeks of age, female TSOD mice over 27 weeks of age, male *db/db* mice over 26 weeks of age, and female *db/db* mice over 32 weeks of age are not used for breeding because pregnancy rates and litter sizes evidently decrease. Compared to young mice, 9-month-old TSOD and *db/db* mice have an evidently lower reproductive capacity. In the future, examinations with longer observational periods using animal models with more detailed data on the reproductive age should be performed. To the best of our knowledge, there has been no report on serum estrogen levels in mice of approximately 30 weeks of age. However, Kubota et al. [30] reported that serum estradiol levels of 50-week-old female 129/Sv mice were lower than those of their 10-week-old counterparts. A large amount of serum is necessary to measure levels of estrogen. In the present study, we were not able to measure serum levels of estrogen since only small amounts of serum were available from mice and we measured the levels of many other serum markers.

Furthermore, we found glucose levels were significantly higher in 3-month-old male *db/db* mice than their female counterparts but significantly higher in 9-month-old female TSOD and *db/db* mice than their male counterparts. As NAFLD is closely associated with diabetes mellitus, this finding may be associated with age-dependent sex differences in NAFLD in these mice. In humans, it has been reported that the prevalence of diabetes is higher in men aged < 60 years and in women at older ages [31]. Therefore, the blood glucose levels in TSOD and *db/db* mice reflect sex-dependent findings in humans. In this study, serum T-Cho, TG, insulin, and adiponectin levels showed significant sex differences in 3- and 9-month-old TSOD and *db/db* mice. However, they did not show age-dependent changes that could explain the severity of NAFLD. Androgens are known to decrease plasma adiponectin [32], and thus, it is reasonable that adiponectin levels were significantly higher in female TSOD mice than in males. However, unexpectedly, adiponectin levels were significantly higher in male *db/db* mice than in females at the age of 3 months. It is difficult to determine the cause of this; however, it has been reported that male, but not female, *db/db* mice show resistance to the effects of adiponectin [33], and this sex-related difference in the adiponectin sensitivity of *db/db* mice might be associated with this finding. CRP is a marker of inflammation, and its levels were remarkably higher in male TSOD and *db/db* mice than in females at 3 months of age. However, at 9 months of age, the sex difference became less remarkable in TSOD mice, and there was no significant sex difference in *db/db* mice. This finding is compatible with the age-dependent sex difference in NAFLD.

In the histopathological analysis, steatosis decreased with age in male *db/db* mice. This finding is consistent with the report by Trayhurn et al. showing that hepatic lipogenesis in *db/db* mice increases progressively from weaning until 8 weeks of age and then decreases [34]. However, in the present study, hepatic steatosis in 3-month-old female *db/db* mice was much milder than that in their male counterparts, and there was no remarkable difference in the extent of steatosis between 3-month-old and 9-month-old female *db/db* mice. However, the extent of fibrosis in *db/db* mice was slightly increased with age for both sexes.

Hepatocyte apoptosis plays a role in the activation of NAFLD/NASH, and apoptotic hepatocytes stimulate immune cells and hepatic stellate cells to initiate the progression of fibrosis in the liver through the production of inflammasomes and cytokines [35]. Hepatocyte apoptosis in TSOD mice was significantly more prevalent in males only at 3 months of age, and this might comprise one of the mechanisms of age-dependent sex difference in NAFLD in this strain. However, positively stained hepatocytes in *db/db* mice showed no significant sex difference in both age groups.

Regarding the biochemical quantification of hepatic TG and T-Cho, both 3-month-old and 9-month-old male *db/db* mice showed significantly higher levels than their female counterparts. However, in the semi-quantitative analysis and quantitative image analysis of histopathology, only 3-month-old male *db/db* mice showed significantly more severe steatosis than females. Thus, there was a discrepancy between biochemical and histopathological data. One of the causes of this might be sampling bias associated with the biochemical analysis since only a small amount of liver tissue (approximately 50 mg for each mouse) was analyzed. In contrast, the largest cut surfaces of the two large liver lobes were analyzed in the histopathological examinations.

Moreover, the hepatic expression levels of *FGF21* mRNA, determined by real-time RT-PCR, were significantly higher in 3-month-old female TSOD mice than their male counterparts and significantly higher in 9-month-old female TSOD and *db/db* mice than males. Serum levels of FGF21 basically reflected the expression levels of *FGF21* mRNA in the liver. FGF21 is known to increase energy expenditure, fat utilization, and lipid excretion, causing weight loss, increased insulin sensitivity, decreased blood glucose and lipid levels, and the amelioration of hepatic steatosis [36–39]. The higher expression of this molecule in female mice than in males may explain male predominance of NAFLD at a young age. However, it was difficult to attribute the age-dependent sex difference in NAFLD to this molecule, since its expression level was significantly higher in old females than males. Lee et al. [14] reported that *FGF21* expression was increased in the liver tissue by the MCD diet, and the degree of upregulation was significantly higher in the livers of female mice. On the other hand, Gasparin et al. [16] reported that *FGF21* mRNA expression was exclusively induced in male mice when mice were fed a cafeteria diet. Therefore, it seems that sex differences in the expression levels of *FGF21* depend on the NAFLD model used. Thus, the role of FGF21 in sex differences in NAFLD should be examined further in the future. MYD88 is an adaptor protein that plays a pivotal role in innate and adaptive immunity. PPARs are nuclear receptors that regulate glucose and lipid metabolism, as well as inflammation [40]. In this study, expression levels of these genes did not show significant sex differences. Expression of *TNF-α* and *IL-6*, representative pro-inflammatory cytokines, was not detectable. In the additional real-time RT-PCR assays, expression of the genes examined was not detectable for many samples even though we tried two different methods. Frozen liver samples were preserved for more than 4 years before the additional real-time RT-PCR experiments, and sample deterioration was probably the cause of this. The samples from 3-month-old mice were preserved longer than those from 9-month-old mice, and the detection of gene expression was especially difficult when using the samples from 3-month-old mice. Although significant sex differences were observed in the expression levels of *FASN* and *CPT 1A*, the interpretation of this result requires caution considering the decreased quality of samples.

Our study has several limitations. Hepatic steatosis in TSOD mice was very mild, and it may be questionable whether TSOD mice fed the MF diet are appropriate as a model of NAFLD. Although remarkable steatosis was observed in the livers of *db/db* mice, necroinflammatory changes were mild, and ballooning degeneration was not conspicuous. This finding conforms to previous observations that *db/db* mice do not spontaneously develop NASH [20,22]. In the future, animal models that better reflect both sex differences and the histopathology of NASH should be developed. In addition, the detailed molecular mechanisms underlying age-dependent sex differences in NAFLD were not elucidated in this study. In the future, extensive molecular studies should be performed using TSOD or *db/db* mice, or further improved animal models. In particular, it is known that clear sex differences exist in immune regulation and response [41], and it would be interesting to examine the associated molecules. Since the present study was performed using mice, only a small amount of serum was available and only a limited number of serum markers could be measured. Especially, serum levels of IL-6, IL-1, and lipopolysaccharide should be examined in the future.

## Conclusions

In this study, serum AST and ALT levels and hepatic steatosis and fibrosis in TSOD and *db/db* mice showed age-dependent sex differences, consistent with those in human NAFLD. These mice may be suitable for studying sex differences of the disease. Elucidation of the detailed mechanisms and development of animal models with more severe histopathological changes should be performed in the future.

## Supporting information

S1 Fig Expression of genes that are associated with lipid metabolism and inflammation in the liver, as determined by additional real-time reverse transcription polymerase chain reaction. **A)** At 3 months, expression levels of *fatty acid synthase (FASN)* are significantly higher in male TSOD mice than in females but are significantly higher in female *db/db* mice than in males (one- or two-tailed t-test). **B)** Expression levels of *carnitine palmitoyltransferase (CPT) 1A* are significantly higher in 9-month-old female TSOD mice than in their male counterparts (two-tailed t-test). **C-F)** For expression levels of *acetyl-CoA carboxylase (ACC)*, *CD36*, *F4/80*, and interleukin (*IL)-1b*, no significant sex difference is detected (one- or two-tailed t-test).
(TIF)

S1 Table Body and liver weights. (XLSX)

S2 Table Serum data of 3-month-old mice. (XLSX)

S3 Table Serum data of 9-month-old mice. (XLSX)

S4 Table Serum insulin levels. (XLSX)

S5 Table Serum adiponectin levels. (XLSX)

S6 Table Liver histopathology of 3-month-old mice. (XLSX)

S7 Table Liver histopathology of 9-month-old mice. (XLSX)

S8 Table Image analysis for steatosis and fibrosis. (XLSX)

S9 Table Expression of *PPARα* and *FGF21* in the liver. (XLSX)

S10 Table Expression of *MYD88* in the liver. (XLSX)

S11 Table Expression of *PPARγ* in the liver. (XLSX)

S12 Table TG levels in the liver. (XLSX)

S13 Table T-Cho levels in the liver. (XLSX)

S14 Table Number of TUNEL stain-positive hepatocytes. (XLSX)

S15 Table Expression of *ACC*, *CD36*, *FASN*, and *CPT 1A* in the liver. (XLSX)

S16 Table Expression of *CD11b*, *CD11c*, *F4/80*, *IL-1b*, and *IL-8* in the liver. (XLSX)

S17 Table Serum CRP levels. (XLSX)

S18 Table Serum TNF-α levels. (XLSX)

S19 Table Serum FGF21 levels. (XLSX)

## References

[1] Charlton M, Kasparova P, Weston S, Lindor K, Maor-Kendler Y, Wiesner RH, et al. Frequency of nonalcoholic steatohepatitis as a cause of advanced liver disease. Liver Transpl. 2001; 7: 608–614. doi: 10.1053/jlts.2001.25453 11460228

[2] Adams LA, Lymp JF, St Sauver J, Sanderson SO, Lindor KD, Feldstein A, et al. The natural history of nonalcoholic fatty liver disease: a population-based cohort study. Gastroenterology. 2005; 129: 113–121. doi: 10.1053/j.gastro.2005.04.014 16012941

[3] Yasui K, Hashimoto E, Komorizono Y, Koike K, Arii S, Imai Y, et al. Characteristics of patients with nonalcoholic steatohepatitis who develop hepatocellular carcinoma. Clin Gastroenterol Hepatol. 2011; 9: 428–433. doi: 10.1016/j.cgh.2011.01.023 21320639

[4] Younossi ZM, Koenig AB, Abdelatif D, Fazel Y, Henry L, Wymer M. Global epidemiology of nonalcoholic fatty liver disease-Meta-analytic assessment of prevalence, incidence, and outcomes. Hepatology. 2016; 64: 73–84. doi: 10.1002/hep.28431 26707365

[5] Blachier M, Leleu H, Peck-Radosavljevic M, Valla DC, Roudot-Thoraval F. The burden of liver disease in Europe: a review of available epidemiological data. J Hepatol. 2013; 58: 593–608. doi: 10.1016/j.jhep.2012.12.005 23419824

[6] Wang Z, Xu M, Hu Z, Hultström M, Lai E. Sex-specific prevalence of fatty liver disease and associated metabolic factors in Wuhan, south central China. Eur J Gastroenterol Hepatol. 2014; 26: 1015–1021. doi: 10.1097/MEG.0000000000000151 25003744

[7] Lonardo A, Nascimbeni F, Ballestri S, Fairweather D, Win S, Than TA, et al. Sex differences in nonalcoholic fatty liver disease: state of the art and identification of research gaps. Hepatology. 2019; 70: 1457–1469. doi: 10.1002/hep.30626 30924946 PMC6766425

[8] Tobari M, Hashimoto E. Characteristic features on nonalcoholic fatty liver disease in Japan with a focus on the roles of age, sex and body mass index. Gut Liver. 2020; 14: 537–545. 31887811 10.5009/gnl19236 PMC7492496

[9] DiStefano JK. NAFLD and NASH in postmenopausal women: implications for diagnosis and treatment. Endocrinology. 2020; 161: bqaa134. doi: 10.1210/endocr/bqaa134 32776116 PMC7473510

[10] Burra P, Bizzaro D, Gonta A, Shalaby S, Gambato M, Morelli MC, et al. Clinical impact of sexual dimorphism in non-alcoholic fatty liver disease (NAFLD) and non-alcoholic steatohepatitis (NASH). Liver Int. 2021; 41: 1713–1733. 33982400 10.1111/liv.14943

[11] Kirsch R, Clarkson V, Shephard EG, Marais DA, Jaffer MA, Woodburne VE, et al. Rodent nutritional model of non-alcoholic steatohepatitis: species, strain and sex difference studies. J Gastroenterol Hepatol. 2003; 18: 1272–1282. doi: 10.1046/j.1440-1746.2003.03198.x 14535984

[12] Anezaki Y, Ohshima S, Ishii H, Kinoshita N, Dohmen T, Kataoka E, et al. Sex difference in the liver of hepatocyte-specific Pten-deficient mice: a model of nonalcoholic steatohepatitis. Hepatol Res. 2009; 39: 609–618. doi: 10.1111/j.1872-034X.2009.00494.x 19527485

[13] Ganz M, Csak T, Szabo G. High fat diet feeding results in gender specific steatohepatitis and inflammasome activation. World J Gastroenterol. 2014; 20: 8525–8534. doi: 10.3748/wjg.v20.i26.8525 25024607 PMC4093702

[14] Lee YH, Kim SH, Kim SN, Kwon HJ, Kim JD, Oh JY, et al. Sex-specific metabolic interactions between liver and adipose tissue in MCD diet-induced non-alcoholic fatty liver disease. Oncotarget. 2016; 7: 46959–46971. doi: 10.18632/oncotarget.10506 27409675 PMC5216916

[15] Spruss A, Henkel J, Kanuri G, Blank D, Püschel GP, Bischoff SC, et al. Female mice are more susceptible to nonalcoholic fatty liver disease: sex-specific regulation of the hepatic AMP-activated protein kinase-plasminogen activator inhibitor 1 cascade, but not the hepatic endotoxin response. Mol Med. 2012; 18: 1346–1355. doi: 10.2119/molmed.2012.00223 22952059 PMC3521787

[16] Gasparin FRS, Carreno FO, Mewes JM, Gilglioni EH, Pagadigorria CLS, Natali MRM, et al. Sex differences in the development of hepatic steatosis in cafeteria diet-induced obesity in young mice. Biochim Biophys Acta Mol Basis Dis. 2018; 1864: 2495–2509. doi: 10.1016/j.bbadis.2018.04.004 29653185

[17] Kashireddy PR, Rao MS. Sex differences in choline-deficient diet-induced steatohepatitis in mice. Exp Biol Med (Maywood). 2004; 229: 158–162. doi: 10.1177/153537020422900204 14734794

[18] Watahiki T, Okada K, Warabi E, Nagaoka T, Suzuki H, Ishige K, et al. Gender difference in development of steatohepatitis in *p62/Sqstm1* and *Nrf2* double-knockout mice. Exp Anim. 2020; 69: 395–406. 32493884 10.1538/expanim.20-0028 PMC7677087

[19] Nishida T, Tsuneyama K, Fujimoto M, Nomoto K, Hayashi S, Miwa S, et al. Spontaneous onset of nonalcoholic steatohepatitis and hepatocellular carcinoma in a mouse model of metabolic syndrome. Lab Invest. 2013; 93: 230–241. doi: 10.1038/labinvest.2012.155 23212097

[20] Takahashi Y, Fukusato T. Animal models of liver diseases. In: Conn PM, editor. Animal models for the study of human disease, 2nd Edition. Cambridge: Academic Press/Elsevier; 2017. pp. 313–339.

[21] Chen H, Charlat O, Tartaglia LA, Woolf EA, Weng X, Ellis SJ, et al. Evidence that the diabetes gene encodes the leptin receptor: identification of a mutation in the leptin receptor gene in *db/db* mice. Cell. 1996; 84: 491–495. 8608603 10.1016/s0092-8674(00)81294-5

[22] Takahashi Y, Soejima Y, Fukusato T. Animal models of nonalcoholic fatty liver disease/nonalcoholic steatohepatitis. World J Gastroenterol. 2012; 18: 2300–2308. doi: 10.3748/wjg.v18.i19.2300 22654421 PMC3353364

[23] Kleiner DE, Brunt EM, Van Natta M, Behling C, Contos MJ, Cummings OW, et al. Design and validation of a histological scoring system for nonalcoholic fatty liver disease. Hepatology. 2005; 41: 1313–1321. doi: 10.1002/hep.20701 15915461

[24] Takahashi Y, Soejima Y, Kumagai A, Watanabe M, Uozaki H, Fukusato T. Inhibitory effects of Japanese herbal medicines sho-saiko-to and juzen-taiho-to on nonalcoholic steatohepatitis in mice. PLoS One. 2014; 9: e87279. doi: 10.1371/journal.pone.0087279 24466347 PMC3899375

[25] Takahashi Y, Watabe S, Togashi-Kumagai A, Watanabe M, Dungubat E, Kusano H, et al. Effects of low ethanol consumption on nonalcoholic steatohepatitis in mice. Alcohol. 2020; 87: 51–61. doi: 10.1016/j.alcohol.2020.04.004 32553943

[26] Forlano R, Mullish BH, Giannakeas N, Maurice JB, Angkathunyakul N, Lloyd J, et al. High-throughput, machine learning-based quantification of steatosis, inflammation, ballooning, and fibrosis in biopsies from patients with nonalcoholic fatty liver disease. Clin Gastroenterol Hepatol. 2020; 18: 2081–2090.e9. doi: 10.1016/j.cgh.2019.12.025 31887451 PMC7397508

[27] De Rudder M, Bouzin C, Nachit M, Louvegny H, Velde GV, Julé Y, et al. Automated computerized image analysis for the user-independent evaluation of disease severity in preclinical models of NAFLD/NASH. Lab Invest. 2020; 100: 147–160. doi: 10.1038/s41374-019-0315-9 31506634

[28] Dungubat E, Watabe S, Togashi-Kumagai A, Watanabe M, Kobayashi Y, Harada N, et al. Effects of caffeine and chlorogenic acid on nonalcoholic steatohepatitis in mice induced by choline-deficient, L-amino acid-defined, high-fat diet. Nutrients. 2020; 12: 3886. doi: 10.3390/nu12123886 33353230 PMC7767129

[29] Sataranatarajan K, Ikeno Y, Bokov A, Feliers D, Yalamanchili H, Lee HJ, et al. Rapamycin increases mortality in *db/db* mice, a mouse model of type 2 diabetes. J Gerontol A Biol Sci Med Sci. 2016; 71: 850–857. 26442901 10.1093/gerona/glv170 PMC4906320

[30] Kubota K, Kubota R, Matsuzawa T. Growth and differentiation of teratocarcinoma OTT6050 affected by host hormonal changes in old mice. Tohoku J Exp Med. 1987; 151: 253–259. doi: 10.1620/tjem.151.253 3590172

[31] Wild S, Roglic G, Green A, Sicree R, King H. Global prevalence of diabetes: estimates for the year 2000 and projections for 2030. Diabetes Care. 2004; 27: 1047–1053. doi: 10.2337/diacare.27.5.1047 15111519

[32] Nishizawa H, Shimomura I, Kishida K, Maeda N, Kuriyama H, Nagaretani H, et al. Androgens decrease plasma adiponectin, an insulin-sensitizing adipocyte-derived protein. Diabetes. 2002; 51: 2734–2741. doi: 10.2337/diabetes.51.9.2734 12196466

[33] Chattopadhyay S, Joharapurkar A, Das N, Khatoon S, Kushwaha S, Gurjar AA, et al. Estradiol overcomes adiponectin-resistance in diabetic mice by regulating skeletal muscle adiponectin receptor 1 expression. Mol Cell Endocrinol. 2022; 540: 111525. doi: 10.1016/j.mce.2021.111525 34856343

[34] Trayhurn P, Wusteman MC. Lipogenesis in genetically diabetic *(db/db)* mice: developmental changes in brown adipose tissue, white adipose tissue and the liver. Biochim Biophys Acta. 1990; 1047: 168–174. doi: 10.1016/0005-2760(90)90043-w 2248973

[35] Kanda T, Matsuoka S, Yamazaki M, Shibata T, Nirei K, Takahashi H, et al. Apoptosis and non-alcoholic fatty liver diseases. World J Gastroenterol. 2018; 24: 2661–2672. doi: 10.3748/wjg.v24.i25.2661 29991872 PMC6034146

[36] Flippo KH, Potthoff MJ. Metabolic messengers: FGF21. Nat Metab. 2021; 3: 309–317. doi: 10.1038/s42255-021-00354-2 33758421 PMC8620721

[37] Coskun T, Bina HA, Schneider MA, Dunbar JD, Hu CC, Chen Y, et al. Fibroblast growth factor 21 corrects obesity in mice. Endocrinology. 2008; 149: 6018–6027. doi: 10.1210/en.2008-0816 18687777

[38] Xu J, Stanislaus S, Chinookoswong N, Lau YY, Hager T, Patel J, et al. Acute glucose-lowering and insulin-sensitizing action of FGF21 in insulin-resistant mouse models–association with liver and adipose tissue effects. Am J Physiol Endocrinol Metab. 2009; 297: E1105–1114. doi: 10.1152/ajpendo.00348.2009 19706786

[39] Xu J, Lloyd DJ, Hale C, Stanislaus S, Chen M, Sivits G, et al. Fibroblast growth factor 21 reverses hepatic steatosis, increases energy expenditure, and improves insulin sensitivity in diet-induced obese mice. Diabetes. 2009; 58: 250–259. doi: 10.2337/db08-0392 18840786 PMC2606881

[40] Fougerat A, Montagner A, Loiseau N, Guillou H, Wahli W. Peroxisome proliferator-activated receptors and their novel ligands as candidates for the treatment of non-alcoholic fatty liver disease. Cells. 2020; 9: 1638. doi: 10.3390/cells9071638 32650421 PMC7408116

[41] He F, Furones AR, Landgren N, Fuxe J, Sarhan D. Sex dimorphism in the tumor microenvironment–from bench to bedside and back. Semin Cancer Biol. 2022; 86(Pt3): 166–179. doi: 10.1016/j.semcancer.2022.03.007 35278635

