# Supplementary figures and images for: Age-dependent sex difference of non-alcoholic fatty liver disease in TSOD and db/db mice
Source: PLoS One. 2022 Dec 14;17(12):e0278580. doi: 10.1371/journal.pone.0278580 (PMC9750023; doi:10.1371/journal.pone.0278580)

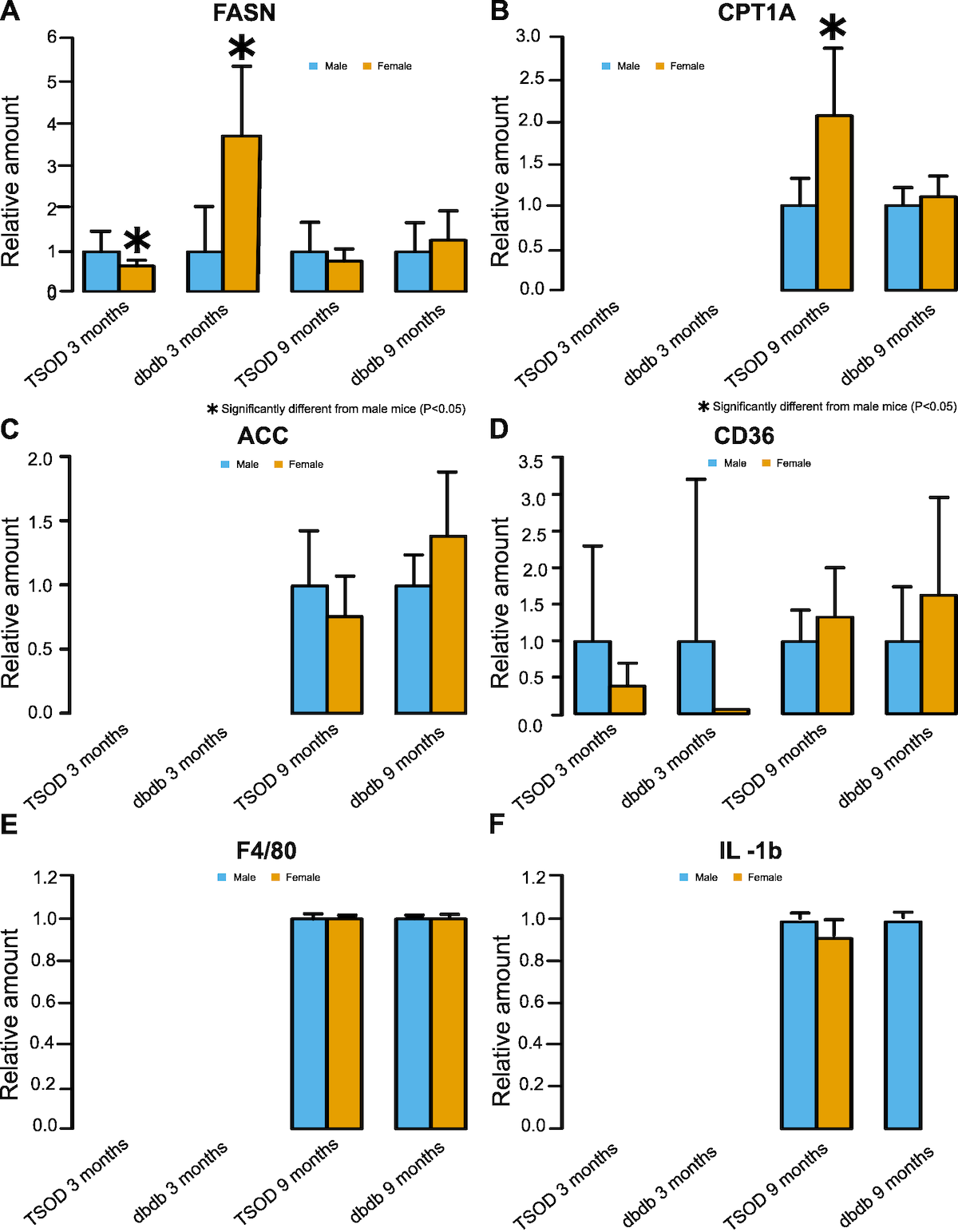

Supplement: S1 Fig — A) At 3 months, expression levels of fatty acid synthase (FASN) are significantly higher in male TSOD mice than in females but are significantly higher in female db/db mice than in males (one- or two-tailed t-test). B) Expression levels of carnitine palmitoyltransferase (CPT) 1A are significantly higher in 9-month-old female TSOD mice than in their male counterparts (two-tailed t-test). C-F) For expression levels of acetyl-CoA carboxylase (ACC), CD36, F4/80, and interleukin (IL)-1b, no significant sex difference is detected (one- or two-tailed t-test). (TIF) [file pone.0278580.s001.tif]
